# Biological control of potato common scab by *Bacillus amyloliquefaciens* Ba01

**DOI:** 10.1371/journal.pone.0196520

**Published:** 2018-04-26

**Authors:** Chih Lin, Chia-Hsin Tsai, Pi-Yu Chen, Chia-Yen Wu, Ya-Lin Chang, Yu-Liang Yang, Ying-Lien Chen

**Affiliations:** 1 Department of Plant Pathology and Microbiology, National Taiwan University, Taipei, Taiwan; 2 Department of Plant Pathology, Taiwan Agricultural Research Institute, Taichung, Taiwan; 3 Agricultural Biotechnology Research Center, Academia Sinica, Taipei, Taiwan; College of Agricultural Sciences, UNITED STATES

## Abstract

Potato common scab, which is caused by soil-borne *Streptomyces* species, is a severe plant disease that results in a significant reduction in the economic value of potatoes worldwide. Due to the lack of efficacious pesticides, crop rotations, and resistant potato cultivars against the disease, we investigated whether biological control can serve as an alternative approach. In this study, multiple *Bacillus* species were isolated from healthy potato tubers, and *Bacillus amyloliquefaciens* Ba01 was chosen for further analyses based on its potency against the potato common scab pathogen *Streptomyces scabies*. Ba01 inhibited the growth and sporulation of *S*. *scabies* and secreted secondary metabolites such as surfactin, iturin A, and fengycin with potential activity against *S*. *scabies* as determined by imaging mass spectrometry. In pot assays, the disease severity of potato common scab decreased from 55.6 ± 11.1% (inoculated with *S*. *scabies* only) to 4.2 ± 1.4% (inoculated with *S*. *scabies* and Ba01). In the field trial, the disease severity of potato common scab was reduced from 14.4 ± 2.9% (naturally occurring) to 5.6 ± 1.1% after Ba01 treatment, representing evidence that *Bacillus* species control potato common scab in nature.

## Introduction

Potato is one of the most important crops worldwide but is easily affected by serious diseases such as late blight, bacterial wilt, soft rot, and common scab. Potato common scab can be caused by at least four gram-positive bacteria from the *Streptomyces* genus, including *S*. *scabies*, *S*. *acidiscabies*, *S*. *turgidiscabies*, and *S*. *ipomoeae*. Of these, *S*. *scabies* is the best characterized pathogen [1, 2]. The typical scab symptom on potato tubers is superficial, raised, deep-pitted corky lesions that affect tuber quality and marketability in fresh markets or processing operations. Scab symptoms are mainly caused by secreted toxins from *Streptomyces* species such as thaxtomins, concanamycin, borrelidin, or FD-891 [1, 3, 4]. Among these, thaxtomin A, a cellulose biosynthesis inhibitor, is well characterized and considered the main virulence factor of most *Streptomyces* species [5].

Because of the limited understanding of the genetic diversity of *S*. *scabies* and the genetic differences in various potato cultivars, developing effective control strategies for potato common scab is challenging [6–10]. Traditional control methods such as soil amendment/chemistry to lower soil pH, soil fumigation with chloropicrin, pre-sowing treatment of seed tubers with fluazinam or flusulfamide, and crop rotation are usually not efficacious and may harm the environment [6, 11, 12]. Research in biological control as an alternative approach is emerging. Several studies have used biocontrol agents, including non-pathogenic *Streptomyces* spp. [13–15], *Pseudomonas* spp. [16–18], and *Bacillus* spp. [19, 20], to combat potato common scab. Currently, only two studies have reported the effects of *Bacillus* spp. on potato common scab. Han *et al*. demonstrated that *Bacillus sp*. sunhua secreted iturin A and macrolactin A as potential antibacterial agents and inhibited the sporulation of *S*. *scabies* [19]. Meng *et al*. showed that *Bacillus amyloliquefaciens* BAC03 secretes LCI protein as an antibacterial product and increases plant height and tuber weight, in addition to reducing the disease severity of potato common scab in pot assays [20]. However, whether these two *Bacillus* species can control the scab pathogen *Streptomyces* species in agricultural fields is unclear.

*Bacillus* species, including *B*. *subtilis* and *B*. *amyloliquefaciens*, produce endospores, resulting in a long shelf life (~2 years), a desirable characteristic for a biocontrol agent. Although *B*. *amyloliquefaciens* is a close relative of *B*. *subtilis*, the secondary metabolites produced by the two species are distinct. For example, *B*. *amyloliquefaciens* FZB42, a commercial strain, dedicates 8.5% of the genome (~340 kb) to synthesize secondary metabolites, which is two-fold higher than that of the *B*. *subtilis* 168 isolate (4.5% of its genome; ~350 kb) [21–24]. Based on genomic analyses, FZB42 can secrete many secondary metabolites, including lipopeptides (surfactin, iturin, and fengycin), polyketides (macrolactin, bacillaene, and difficidin), and volatiles (acetoin/2,3-butandiol), which may directly suppress the growth of plant pathogens or elicit induced systemic resistance (ISR) of the plant host.

In this study, we isolated a biocontrol agent, *B*. *amyloliquefaciens* Ba01, from healthy potato tubers and showed its inhibitory effects on the growth and sporulation of the potato common scab pathogen *S*. *scabies*. The potential inhibitory mechanism of Ba01 against *S*. *scabies* is possibly through the secretion of lipopeptides such as surfactin, iturin A, and fengycin. Ba01 not only reduced the disease severity of potato common scab in pot assays, but also in scab naturally occurring in the field, representing a documented example that *Bacillus* species reduce the disease severity of potato common scab in nature.

## Materials and methods

### Strains, growth media, chemicals, and phylogenetic analysis

*Bacillus* species and *S*. *scabies* strains are listed in Table 1. *Bacillus* species isolated from healthy potato tubers with nutrient agar medium were identified by sequencing 16S rRNA with primers fD1 and rP2 [25] (Table A in S1 File) and the *gyrase A* gene with primers p-*gyr*A-F and p-*gyr*A-R [26] (Table A in S1 File). *S*. *scabies* strains isolated from scabby potato tubers with nutrient agar or SCNA medium were identified by sequencing 16S rDNA with primers fD1 and rP2 and by PCR-RFLP with amplification of the partial *atpD* gene with primers atpDPF and atpDPR [27] (Table A in S1 File). The sequence results were compared in the Basic Local Alignment Search Tool (http://blast.ncbi.nlm.nih.gov/Blast.cgi). Sequence alignment and analysis of gene similarity were performed using the ClustalW program in the MEGA 7 program. The evolutionary history was inferred by using the Maximum Likelihood method based on the Tamura-Nei model [28]. Phylogenetic trees were drawn using the MEGA7 program [29].

**Table 1:** Strains used in this study.

Strain	Species	Source
Ba01	*Bacillus amyloliquefaciens*	Houli, Taiwan
Ba02	*Bacillus amyloliquefaciens*	Houli, Taiwan
Ba03	*Bacillus amyloliquefaciens*	Houli, Taiwan
Ba04	*Bacillus amyloliquefaciens*	Houli, Taiwan
Bs01	*Bacillus subtilis*	Douliu, Taiwan
*B*. *sp*.	*Bacillus sp*.	Dounan, Taiwan
PS01	*Streptomyces scabies*	[41]
PS02	*Streptomyces scabies*	[41]
PS07	*Streptomyces scabies*	[41]
PS08	*Streptomyces scabies*	[41]
YC1020	*Streptomyces scabies*	[41]
YC1028	*Streptomyces scabies*	[41]
A3(2)	*Streptomyces coelicolor*	[42]
CL2	*Streptomyces scabies*	Dounan, Taiwan
CL3	*Streptomyces scabies*	Dounan, Taiwan
CL4	*Streptomyces scabies*	Dounan, Taiwan
CL5	*Streptomyces scabies*	Dounan, Taiwan

Media used in this study include Nutrient Agar medium (NA: HIMEDIA, India), Starch Casein Nitrate Agar medium (SCNA; 0.001% FeSO_4_7.H_2_O, 0.002% CaCO_3_, 0.005% MgSO_4_.7H_2_O, 0.03% casein, 0.2% K_2_HPO_4_, 0.2% NaCl, 0.2% KNO_3_, 1% starch, and 1.8% agar), Yeast Malt Extract agar medium (YME medium; 0.4% yeast extract, 1% malt extract, 0.4% dextrose, and 2% agar), *Streptomyces* Sporulation Medium #1 (SSM1; 0.1% MgSO_4_.7H_2_O, 0.1% casein, 0.1% yeast extract, 1% soluble starch, 0.05% K_2_HPO_4_, and 2% agar), Luria-Bertani medium (LB; MDBio, Inc., Taiwan), and Mueller Hinton medium (Becton, Dickinson and Company, Franklin Lakes, NJ, USA). The following chemical agents were used in this study: iturin A (Sigma-SI-I1774, St Louis, MO, USA), surfactin (Sigma-SI-S3523), and fengycin (Sigma-SMB00292).

### Disk diffusion assays

Disk diffusion assays were used to test anti-*S*. *scabies* activity of six *Bacillus* strains, including four *B*. *amyloliquefaciens* isolates, one *B*. *subtilis* isolate, and one *Bacillus sp*. isolate. *Bacillus* species were grown overnight at 37°C in LB liquid medium, and the cell concentrations were calculated by measuring OD_600_ (1 OD_600_ = ~2x10^7^ CFU/mL). *S*. *scabies* isolates were grown on solid SSM1 medium at 28°C for 14 days. Spores were collected with cell scrapers and washed twice with ddH_2_O before determining spore concentration by serial dilution. Then, 100 μL of 10^7^ CFU/mL *S*. *scabies* cells were spread on YME solid medium, and two 6-mm disks were placed on the surface of each agar plate. Then, 3 μL of 1 OD_600_ *Bacillus* cells or ddH_2_O were placed on the disk, and plates were cultured at 28°C for five days before being photographed.

### Determination of minimum and fractional inhibitory concentrations

Minimum inhibitory concentration (MIC) indices were determined following the CLSI-M07-A9 protocol. Freshly collected spores of *S*. *scabies* PS07 were diluted with Mueller Hinton medium, and 50 μL were added to each well to a final concentration at 5x10^5^ CFU/mL in a 96-well plate format. The compounds were two-fold serially diluted with Mueller Hinton medium, and 50 μL of each dilution were added to the wells containing spores, yielding a total volume of 100 μL per well. Iturin A concentrations ranged from 0.125 to 64 μg/mL, while surfactin and fengycin concentrations ranged from 0.25 to 64 μg/mL. The plates were incubated at 28°C for 48 h. The MIC was defined as the lowest concentration of a compound that completely inhibited the growth of *S*. *scabies* PS07 as detected by the unaided eye.

The fractional inhibitory concentration (FIC) of compounds was determined via checkerboard titration assays. Freshly collected spores of *S*. *scabies* PS07 were diluted with Mueller Hinton medium, and 50 μL were added to each well of a 96-well plate to a final concentration of 5x10^5^ CFU/mL. The two compounds to be assessed were two-fold serially diluted with Mueller Hinton medium, and 25 μL of each compound were added to the wells containing spores, yielding a total volume of 100 μL per well. Iturin A concentrations ranged from 1 to 64 μg/mL, while surfactin and fengycin concentrations ranged from 0.25 to 64 μg/mL. The plates were incubated at 28°C for 48 h. The MIC or FIC of compounds, either alone or in combination, was defined as the lowest concentration of each compound that completely inhibited the growth of *S*. *scabies* PS07 as detected by the unaided eye. The FIC index was calculated by the following formula: FIC = (MIC of compound A combined) / (MIC of compound A alone) + (MIC of compound B combined) / (MIC of compound B alone). For calculation purposes, an MIC >64 μg/mL was assumed to be 128 μg/mL.

### Scanning electron microscopy

The agar plates from the disk diffusion assay were cultured at 25°C for five days, and the undifferentiating and non-inhibition zones were excised and fixed in 2% glutaraldehyde in 0.1 M sodium cacodylate buffer (pH 7.4) at 4°C for 24 h. The samples were then rinsed three times with cold 0.1 M sodium cacodylate buffer (pH 7.4) for 1 h each time, soaked in 2% osmium tetroxide in sodium cacodylate buffer at 4°C for 5 h, and rinsed again. After fixation, the samples were dehydrated in a series of ethanol concentrations (50%, 70%, 80%, 90%, and 95%) at 4°C for 10 min each and in 100% acetone twice (first at 4°C for 15 min and then at room temperature for 15 min). The samples were then critical point dried in liquid CO_2_, mounted on metal stubs for gold coating, and observed under the scanning electron microscope JEOL JSM 6510 at 15 kV.

### Imaging mass spectrometry

*S*. *scabies* PS07 was initially inoculated in a vertical line on 2% YME solid agar plates. After 12 h, *B*. *amyloliquefaciens* Ba01 was inoculated in a horizontal line on the same plate for an additional 24 h, and the region in which the two microbes interacted, as well as the individual regions with compounds secreted from each microbe, were excised and transferred to an indium tin oxide-coated glass target plate. Pure, serially diluted surfactin, iturin A, and fengycin were used as standards to determine the compound concentration secreted by Ba01. Surfactin and fengycin were two-fold serially diluted with methanol from 2,500 μg/mL to 39.1 μg/mL and from 312.5 μg/mL to 4.9 μg/mL, respectively, while iturin A was two-fold serially diluted with 50% ethanol from 10,000 μg/mL to 156.3 μg/mL. Then, 1 μL of each diluted compound was dropped onto the YME agar. The universal matrix powder (1:1 mixture of α-cyano-4-hydroxycinnamic acid and 2, 5-dihydroxybenzoic acid) was sprinkled on the top of samples. After covering with matrix, the samples were exposed to air overnight at 37°C until dried completely.

Imaging mass spectrometry (IMS) data were collected on a Bruker Autoflex Speed MALDI TOF/TOF spectrometer at the Agricultural Biotechnology Research Center, Academia Sinica and analyzed by Bruker Compass Version 1.2 Software Suite [30]. For the samples used in this study, linear positive ion mode was applied with 85% laser power and 333.3 Hz laser frequency. The 1,100 μm of raster interval in the X and Y dimensions were applied, and each raster summed up to 500 shots. The detection mass range was set from *m/z* 100 to 2125.

### Pot assays

Pot assays were conducted to examine the biocontrol activity of Ba01 against *S*. *scabies* PS07. *S*. *scabies* was grown in solid SSM1 medium at 25°C for 14 days, and the spores were collected by cell scrapers and washed twice with ddH_2_O. Potato (cultivar: Kennebec) tuber pieces with a bud were air dried and planted in nursery pots filled with sterilized soil (1:1 peat moss:King Root plant substrate) at 25°C for three weeks until seedlings emerged. Each potato seedling was transferred to a seven-inch pot in a greenhouse with the temperature maintained between 18°C and 22°C. Five-week-old potato plants were then inoculated with *S*. *scabies* PS07 by mixing 50 mL (2x10^9^ CFU/mL) of inoculum with the soil. For *B*. *amyloliquefaciens* Ba01 treatment, Ba01 was grown overnight in LB liquid medium at 37°C and rinsed twice with ddH_2_O. Then, 50 mL containing 2.8 x10^9^ Ba01 cells were applied to the treatments. Each of the five treatments included three potato plants, for a total of 15 plants: (a) mock control without PS07 or Ba01; (b) PS07 only; (c) Ba01 only; (d) PS07 and Ba01 inoculated on the same day; and (e) PS07 inoculated first for 14 days, followed by inoculation of Ba01. Potato plants were watered twice weekly, and fertilizer (HYPONeX 2) was applied weekly beginning the fifth week after planting. Potato tubers were harvested 12 weeks after planting, and the disease severity in each treatment was calculated as follows, originally described by Wanner *et al*. [13]: Σ (percentage coverage by lesions x predominant lesion type x number of tubers with these scores) / (18 x total number of potato tubers evaluated) x 100. Lesion types were divided into four degrees: 0 = no symptoms, 1 = superficial, 2 = raised, and 3 = pitted. Percentage coverage by lesions for each tuber was classified in seven degrees: 0 = no scab, 1 = 0.1% to 2%, 2 = 2.1% to 5%, 3 = 5.1% to 10%, 4 = 10.1% to 25%, 5 = 25.1% to 50%, and 6 = ≥50%. Disease incidence was calculated by determining the proportion of tubers with >5% scab coverage from collected potato tubers.

### Ethics statement

The field trial was conducted on private land owned by Mr. Rong Su, who permitted and assisted us in performing the biological control experiments using *B*. *amyloliquefaciens* Ba01 against potato common scab. Because the land was privately owned, specific permission was not required. This land did not house endangered or protected species.

### Field trial

The 11-week trial was conducted in an agricultural field with naturally occurring potato common scab from December 2, 2015 to February 17, 2016 in Dounan, Taiwan (23.669919, 120.463699). The temperature in Dounan was between 15°C and 21°C during the experiment. The field was divided into 16 blocks (four blocks per treatment) with a randomized complete block design. Each block contained 20 potato plants, and 10 protective potato plants were located between blocks. Treatments were as follows: (A) 5x10^6^ CFU/mL Ba01, (B) 1x10^7^ CFU/mL Ba01, (C) 2x10^7^ CFU/mL Ba01, and (D) mock (no Ba01). Ba01 was applied at 0, 2, and 3 weeks after planting. The Ba01 cells in a volume of 200 mL were poured directly onto the soil that covered the tuber buds at week 0, and 400 mL of Ba01 were applied 2 and 3 weeks after planting. We randomly collected 25 potato tubers from each block to evaluate the disease severity and incidence of each block, and four blocks of the same treatment were used to determine the mean ± standard error and compared to other treatments based on Tukey’s test.

### Tuber slice assays

Tuber slice assays were used to test the pathogenicity of multiple *S*. *scabies* strains isolated from the field trial, as well as the biocontrol activity of Ba01 against these *S*. *scabies* strains. The procedures used have been described previously by Loria *et al*. [31], with minor modifications. In brief, the surface of potato tuber was sterilized with 1% NaOCl and cores (1.2 cm) of pith tissue were removed from the tubers. The cores were then sliced into pieces (0.25 cm thick) and placed on moist filter paper in glass petri dishes. Three tuber pieces were used for each treatment. Test strains were grown on solid SSM1 medium for 14 days at 28°C, and agar disks with the sporulating colony were inverted onto the tuber pieces. Tuber pieces were incubated in moist glass petri dishes at 28°C for six days in the dark and photographed.

## Results

### *B*. *amyloliquefaciens* inhibited the growth and sporulation of *S*. *scabies*

**Fig 1:**
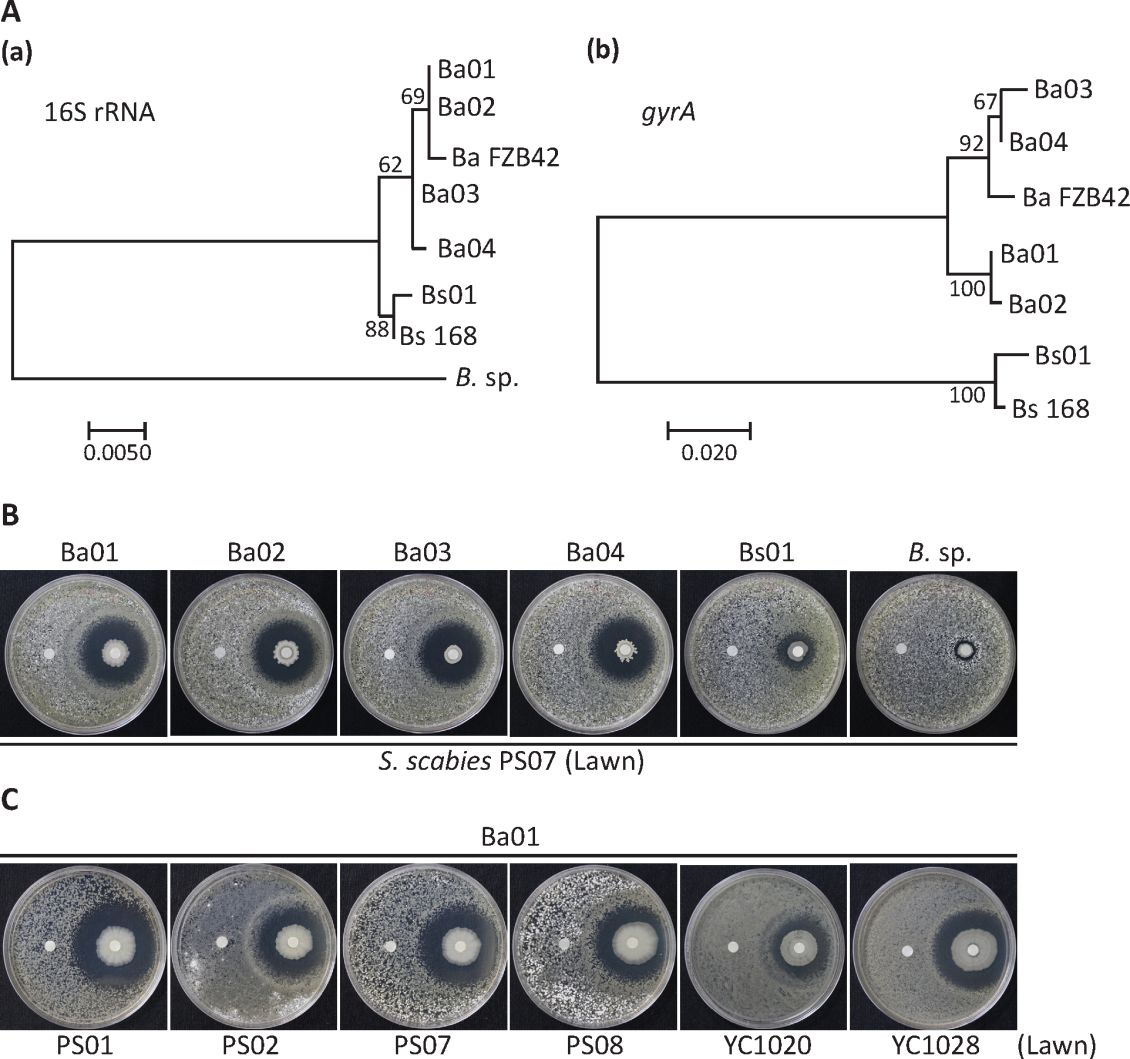
*B*. *amyloliquefaciens* Ba01 showed antibacterial activity against *S*. *scabies* causing potato common scab. A. Phylogenetic trees based on either the (a) 16S rRNA sequence or (b) *gyrA* gene sequence showed the evolutionary relationships between *Bacillus* isolates. The numbers at the nodes represent bootstrap values. The scale bars indicating the numbers of substitutions per nucleotide position were (a) 0.005 and (b) 0.02. **B.** Multiple *Bacillus* isolates exhibited a diverse degree of antibacterial activity against *S*. *scabies* PS07. Disk diffusion assays were used to test the antibacterial activity of *Bacillus* species against *S*. *scabies*. In this experiment, 10^6^ *S*. *scabies* spores in 100 μL were spread on solid YME medium, and 3 μL of 1 OD_600_ (~6x10^4^ cells) of *Bacillus* isolates were loaded on the right disk, while 3 μL of ddH_2_O were loaded on the left disk as a control. **C.** Ba01 was selected to test its antibacterial activity against multiple *S*. *scabies* isolates. *S*. *scabies* isolates were spread on solid YME medium, and 3 μL of 1 OD_600_ of Ba01 were added to the right disk and ddH_2_O to the left disk. All plates were incubated at 28°C for five days and photographed.

**Fig 2:**
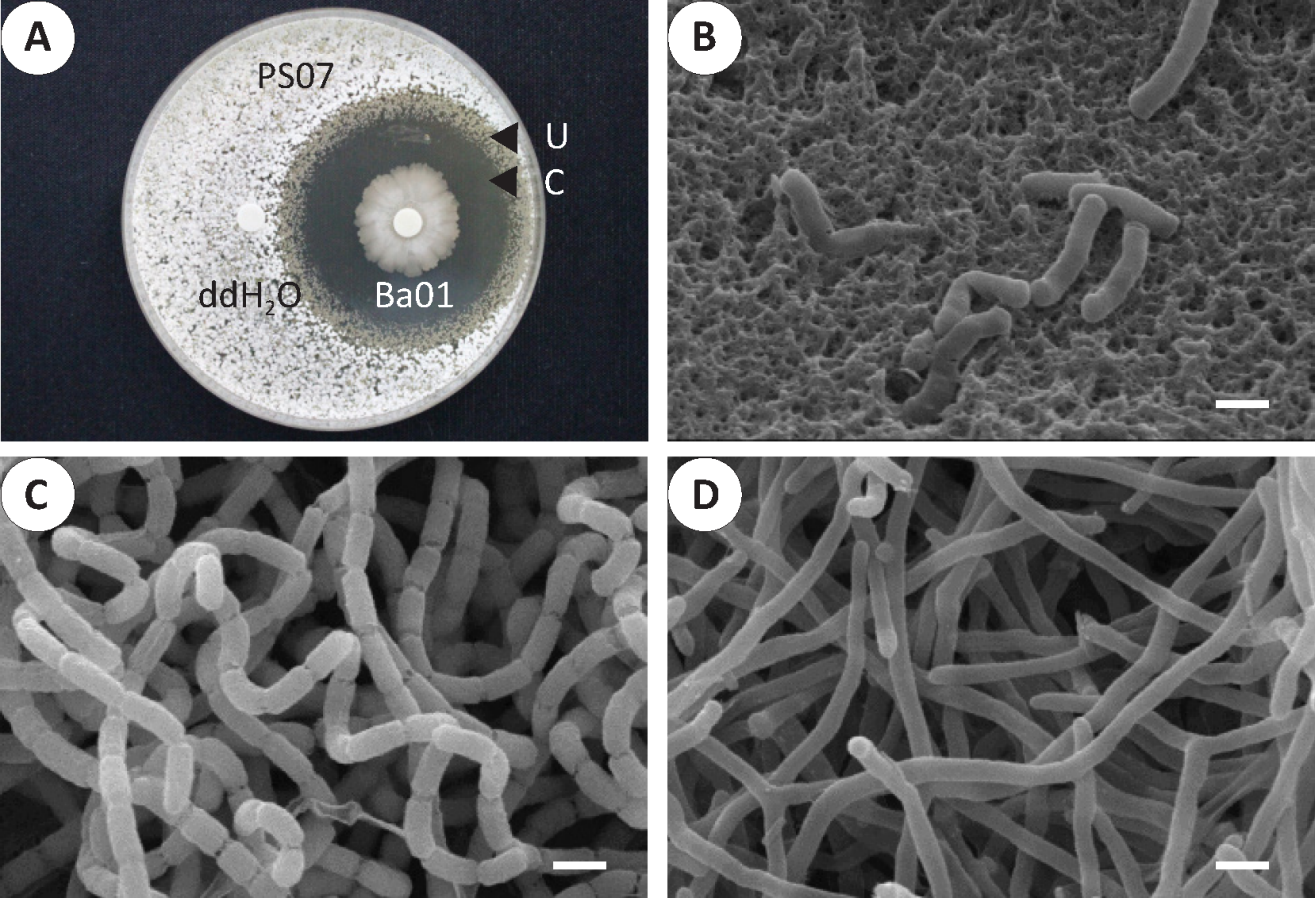
*B*. *amyloliquefaciens* Ba01 inhibited the growth and sporulation of *S*. *scabies* PS07. **A.** A disk diffusion assay was used to observe the antibacterial effects of Ba01 against *S*. *scabies* PS07 on solid YME medium. Clear (C) and undifferentiating (U) zones were observed around the disk loaded with the Ba01 isolate. **B.** The morphology of Ba01 from panel A was observed under a scanning electron microscope. **C.** *S*. *scabies* PS07 without Ba01 treatment (ddH_2_O treated) produced spiral hyphae and sporulation septa with constrictions. **D.** *S*. *scabies* PS07 in the undifferentiating zone exhibited vegetative/smooth hyphae and septa without constrictions. Resolution is 10,000X. The scale bar represents 1 μm.

*Bacillus* strains, including four *B*. *amyloliquefaciens* isolates (Ba01, Ba02, Ba03, and Ba04), a *B*. *subtilis* (Bs01) isolate, and a *Bacillus sp*. isolate with species unidentified were used to test anti-*S*. *scabies* activity *in vitro*. The 16S rRNA and *gyrA* gene sequences of *Bacillus* strains were analyzed to determine phylogenetic assignments. Ba01 clustered closely with Ba02, and Ba03 clustered closely with Ba04. Bs01 and *Bacillus sp*. strains were distinct from Ba01~04 (Fig 1A). In the disk diffusion assays, Ba01, Ba02, Ba03, and Ba04 effectively inhibited the growth of *S*. *scabies* PS07, while *B*. *subtilis* Bs01 and *Bacillus* sp. demonstrated subtle inhibition (Fig 1B). Due to the similar activity of the four *B*. *amyloliquefaciens* isolates, we chose Ba01 to conduct further experiments. We tested the antibacterial activity of Ba01 against multiple *S*. *scabies* isolates (Fig 1C) and found that clear and undifferentiating inhibition zones were formed (Fig 2A and 2B). Based on morphologic observations from scanning electron microscopy, *S*. *scabies* PS07 hyphae were spiral and formed sporulation septa with constrictions (Fig 2C), while hyphae in the undifferentiating zone displayed vegetative septa without constrictions (Fig 2D).

### Identification of secondary metabolites secreted from Ba01 that potentially inhibited *S*. *scabies* growth

**Fig 3:**
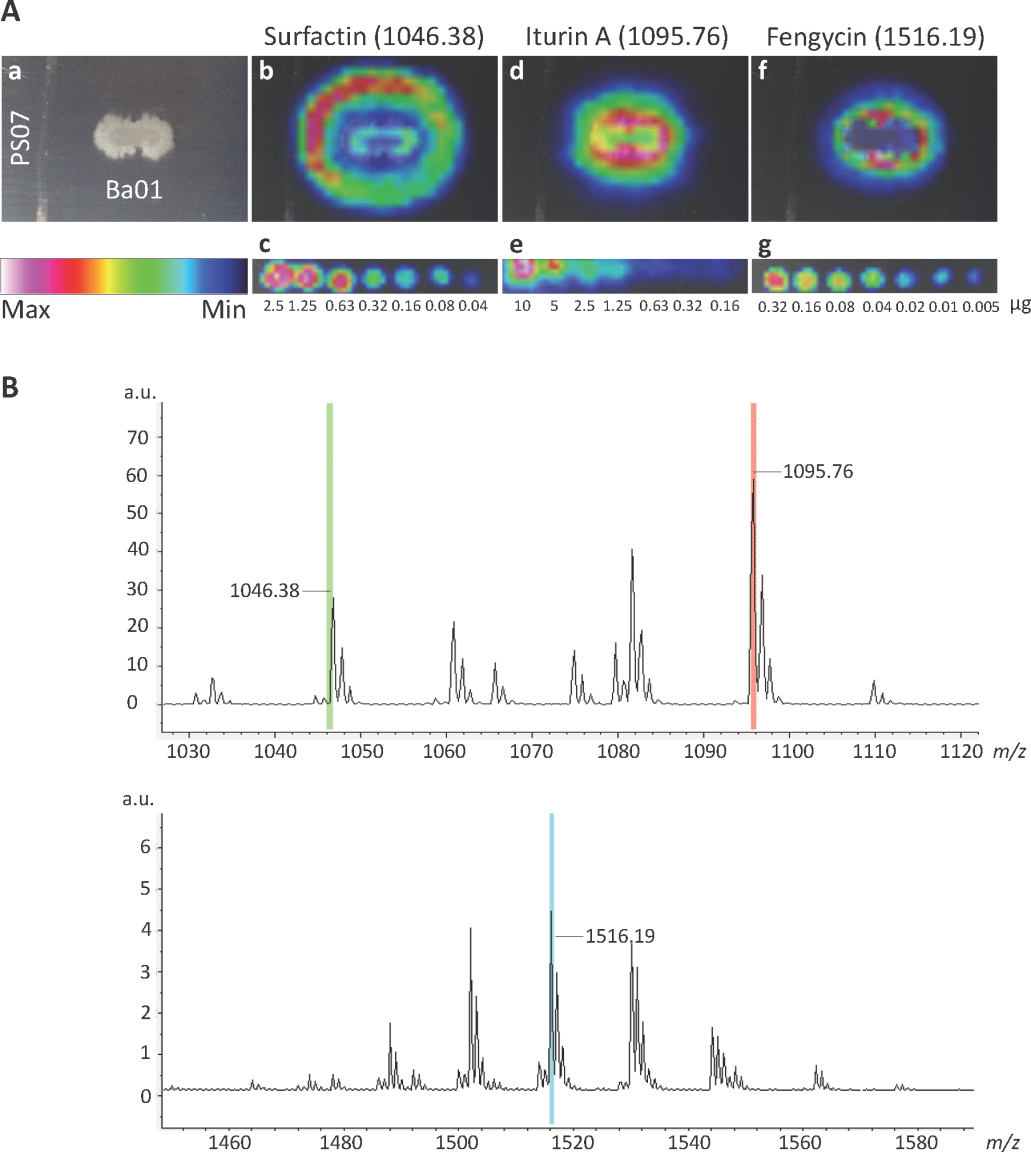
Imaging mass spectrometry of Ba01 against *S*. *scabies* PS07. **A.** (a) *S*. *scabies* PS07 was initially inoculated in a vertical line on a 2% YME solid agar plate. After 12 h, *B*. *amyloliquefaciens* Ba01 was inoculated in a horizontal line on the same plate for another 24 h. (b) The IMS image of an ion with *m/z* 1046.38 represents surfactin. (c) The image represents two-fold serial diluted surfactin as a standard control ranging from 2.5 to 0.04 μg. (d) The IMS image of an ion with *m/z* 1095.76 represents iturin A. (e) The image represents two-fold serial diluted iturin A as a standard control ranging from 10 to 0.16 μg. (f) The IMS image of an ion with *m/z* 1516.19 represents fengycin. (g) The image represents two-fold serial diluted fengycin as a standard control ranging from 0.32 to 0.005 μg/mL. Intensity gradients for surfactin, iturin A, and fengycin are normalized and illustrated by color histogram (maximum, white; minimum, black). **B.** The mass spectra of IMS regions include three major peaks: *m/z* 1046.38 (surfactin), 1095.76 (iturin A), and 1516.19 (fengycin).

**Fig 4:**
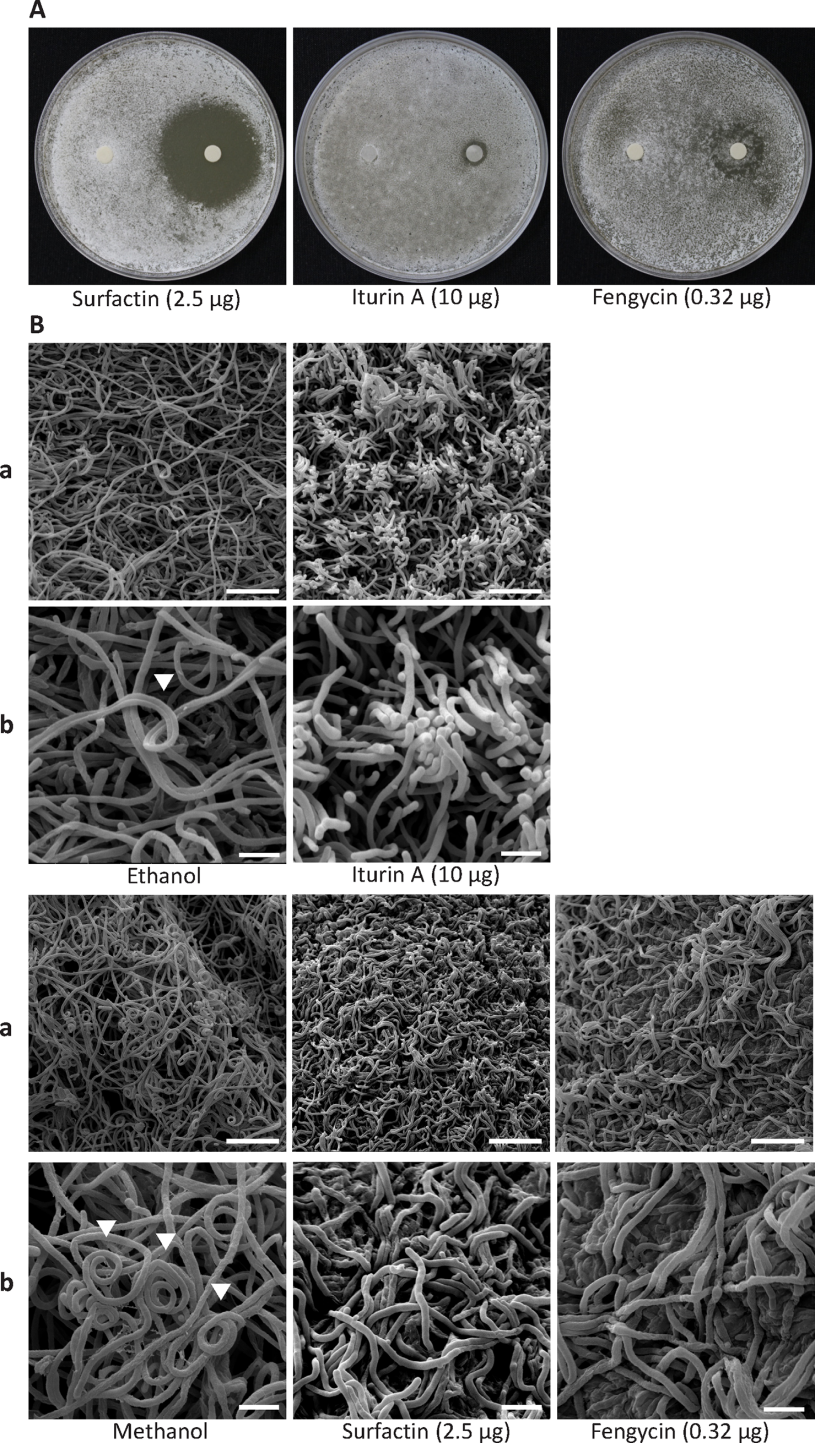
Surfactin, iturin A, and fengycin inhibited the growth and formation of spiral hyphae of *S*. *scabies* PS07. **A.** Disk diffusion assays were used to test the anti-*S*. *scabies* activity of surfactin, iturin A, and fengycin. Approximately 10^6^ *S*. *scabies* spores were spread on YME solid agar, and a 6-mm disk containing iturin A (dissolved in ethanol), surfactin (dissolved in methanol), or fengycin (dissolved in methanol) were pressed on the surface of an agar plate and incubated at 28°C for five days. **B.** The morphology of *S*. *scabies* PS07 in the undifferentiating zone of panel A was observed under a scanning electron microscope. (a) The magnification is 5,000X, and the scale bar represents 10 μm. (b) The magnification is 15,000X, and the scale bar represents 2.5 μm.

IMS is a powerful technique used to visualize the spatial distribution of various chemical compounds based on their molecular masses (e.g., *m/z* ratio) [30]. We used this technique to identify potential secondary metabolites secreted from Ba01 while it inhibited the growth of *S*. *scabies* PS07 on YME solid medium (Fig 3A). Three major peaks representing surfactin (*m/z* 1046.38), iturin A (m/z 1095.76), and fengycin (m/z 1516.19) were detected in the region in which Ba01 inhibited the growth of *S*. *scabies*. Of these, surfactin was secreted largely to neighboring regions at a maximum amount of 2.5 μg, as evidenced by density gradient and IMS spectra. Iturin A was secreted at the strongest intensity with a maximum amount of 10 μg, while fengycin was secreted at a maximum amount of 0.32 μg (Fig 3A and 3B). In order to confirm our findings, pure iturin A, surfactin, and fengycin were used to test the anti-*S*. *scabies* activity. Each of the three compounds demonstrated anti-*S*. *scabies* activity as evidenced by undifferentiating inhibition zones, but none of the compounds completely inhibited the growth of *S*. *scabies* (Fig 4A). Surfactin (2.5 μg) exhibited better inhibitory effects on the growth of *S*. *scabies* than iturin A (10 μg) or fengycin (0.32 μg) based on the disk diffusion assays (Fig 4A). Meanwhile, *S*. *scabies* treated with surfactin, iturin A, or fengycin exhibited defects in the formation of spiral hyphae, while *S*. *scabies* treated with iturin A demonstrated additional morphological defects such as clumped hyphae (Fig 4B). These results suggested that these three compounds identified by IMS inhibit the growth and differentiation of *S*. *scabies*. However, each compound was determined to have a MIC value >64 μg/mL against *S*. *scabies* (Table 2). The contrasting results between the disk diffusion assays (i.e., partial inhibition [undifferentiating] zone on solid medium) and the high MIC values of surfactin, iturin A, and fengycin in liquid medium against *S*. *scabies* might be because the definition of MIC endpoint requires the complete inhibition of growth, whereas some growth persisted even with extensive inhibition. Further experiments were performed to test if these three compounds demonstrated synergistic activity against *S*. *scabies*. However, synergistic activity was not detected based on determined FICs of 2, suggesting no interaction between compounds (Table 2).

**Table 2:** Minimum and fractional inhibitory concentrations of compounds against *Streptomyces scabies* PS07.

Strain	MIC alone (μg/mL)			MIC combined (μg/mL)			*FIC index		
	Iturin A	Surfactin	Fengycin	Iturin A, Surfactin	Iturin A, Fengycin	Surfactin, Fengycin	Iturin A + Surfactin	Iturin A + Fengycin	Surfactin + Fengycin
PS07	>64	>64	>64	>64, >64	>64, >64	>64, >64	2	2	2

*FIC ≤0.5 (synergy); FIC >0.5 but ≤4 (no interaction); FIC >4 (antagonism)

### Ba01 reduced the disease severity of potato common scab in pot assays

**Fig 5:**
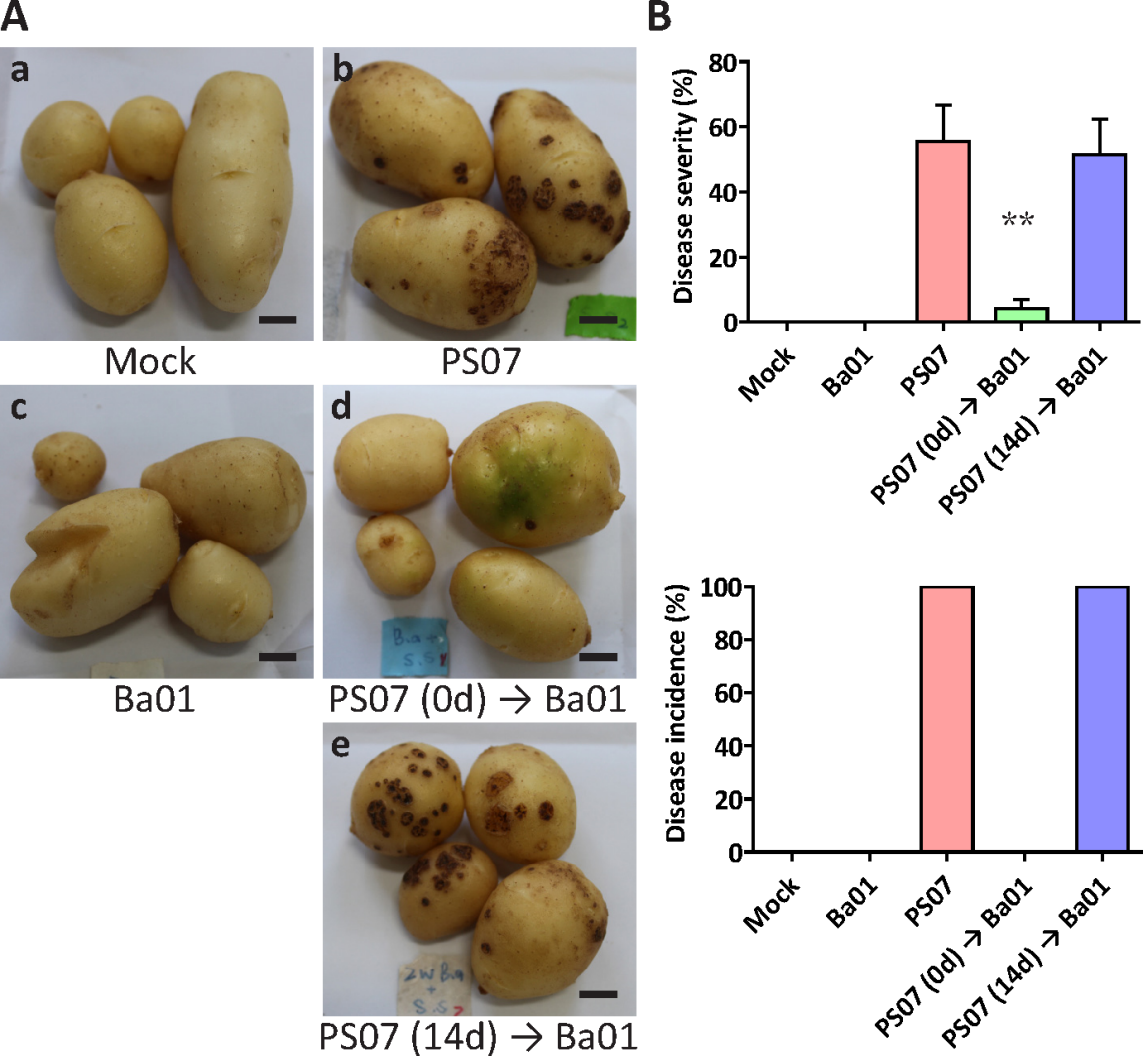
Ba01 reduced the disease severity of potato common scab in pot assays. The growth conditions of *S*. *scabies* PS07, Ba01, and potato plants were described in the materials and methods. **A.** Three five-week-old potato plants were used for each treatment: (a) mock control without inoculation of *S*. *scabies* PS07 or Ba01; (b) inoculation of *S*. *scabies* PS07 only; (c) inoculation of Ba01 only; (d) inoculation of PS07 and Ba01 on the same day; and (e) inoculation of PS07 first, and then Ba01 inoculation after 14 days. The bar represents 1 cm. **B.** The disease severity and disease incidence of the potato common scab were reduced when potato plants were inoculated with PS07 and Ba01 on the same day. Data were expressed as the average of tubers collected from three potato plants ± standard error of the mean. *P* values were calculated using Tukey's test. Asterisks (**) indicate *P* < 0.01 as compared to the treatment of PS07 only.

Pot assays were performed to test whether Ba01 can reduce the disease severity of potato common scab. Fifteen potato plants were divided into five treatments: (a) mock control without PS07 or Ba01; (b) PS07 only; (c) Ba01 only; (d) PS07 and Ba01 inoculated on the same day; and (e) inoculation of PS07 first, then Ba01 inoculation after 14 days. Potato tubers were harvested 12 weeks after planting, and the disease severity was calculated based on an index of percentage coverage by lesions multiplied by the index of predominant lesion type, divided by 18 [13]. Treatment with Ba01 reduced the disease severity of potato common scab from 55.6 ± 11.1% (inoculated with *S*. *scabies* only) to 4.2 ± 1.4% (inoculated with *S*. *scabies* and Ba01 on the same day) (Fig 5A and 5B) (*P* < 0.01, Tukey’s test). However, when Ba01 was applied two weeks after *S*. *scabies* inoculation, it did not reduce the disease severity or incidence (Fig 5A and 5B), indicating that Ba01 demonstrated preventive rather than therapeutic activity.

### Ba01 reduced the disease severity of potato common scab in the field

**Fig 6:**
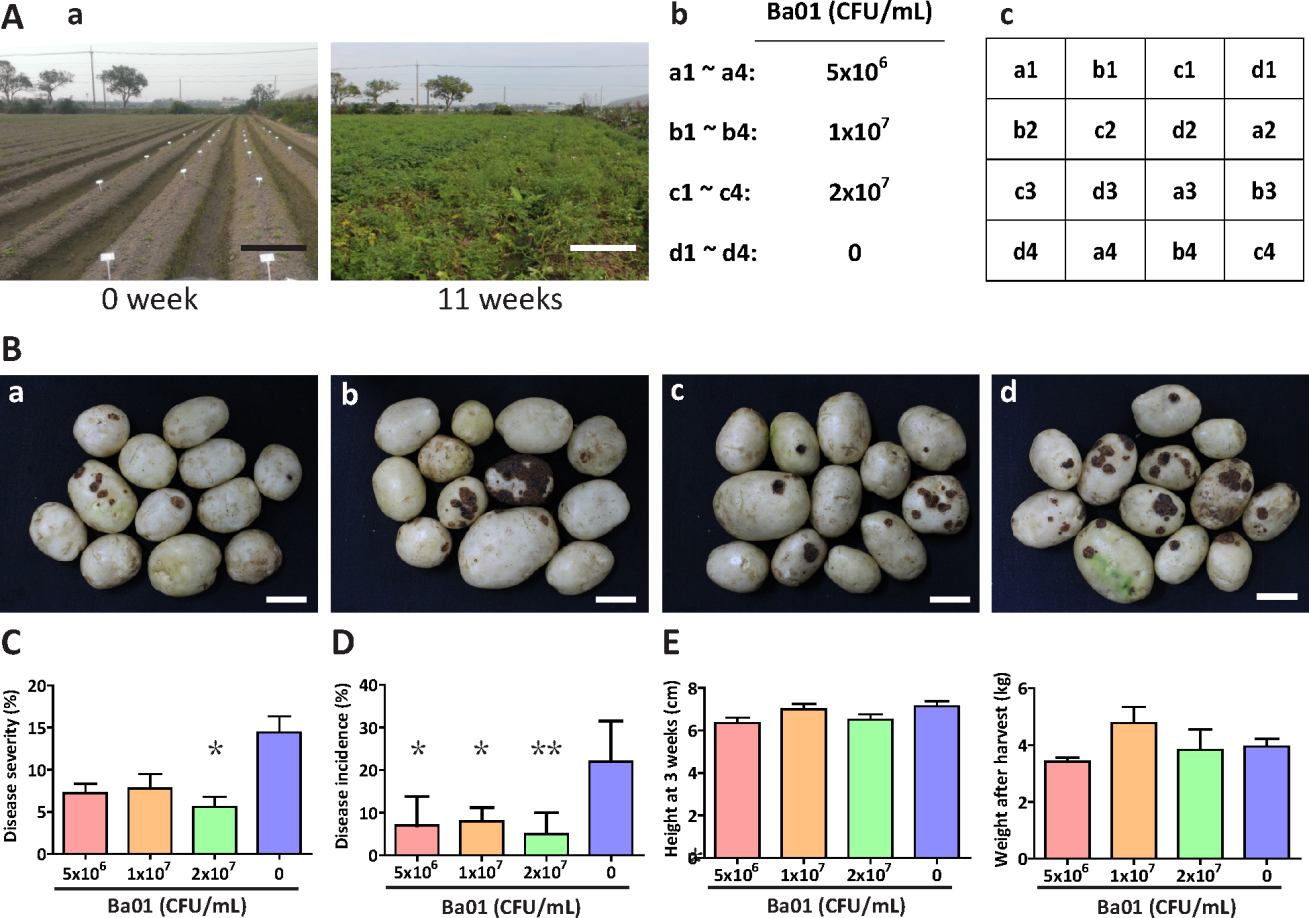
Ba01 reduced the severity of naturally occurring potato common scab in the field. **A.** (a) An 11-week potato field trial was completed in Dounan, Taiwan. Bars = 100 cm. (b) Four treatments were treated with the Ba01 isolate at the concentrations indicated. (c) Each treatment had four blocks assigned by a randomized complete block design. **B.** Potato tubers were harvested from each Ba01 treatment: (1) Ba01 at 5x10^6^ CFU/mL; (b) 1x10^7^ CFU/mL; (c) 2x10^7^ CFU/mL; and (d) water. Bars = 5 cm. **C.** The percentage of disease severity was calculated from 100 randomly selected tubers of each treatment. **D.** Disease incidence was calculated by determining the proportion of tubers with >5% scab coverage from 100 randomly selected tubers in each treatment. **E.** Potato plant height (left panel) and tuber weight (right panel) were not affected by Ba01 application. We randomly chose 25 potato tubers from each block to evaluate the disease severity and incidence of each block, and four blocks of the same treatment were used to determine the mean ± standard error and compare to other treatments. *P* values were calculated using Tukey's test. Asterisks *, **, and *** represent *P* < 0.05, *P* < 0.01, and *P* < 0.001, respectively.

A field trial was conducted in an agricultural field with naturally occurring potato common scab (Fig 6A). Three treatments of Ba01 at 5x10^6^, 1x10^7^, and 2x10^7^ CFU/mL, in addition to a mock control of 0 CFU/mL, in a randomized complete block design were used to test inhibitory activity against potato common scab disease (Fig 6A). Ba01 treatment at 2x10^7^ CFU/mL significantly reduced disease severity from 14.4 ± 1.9% (no Ba01 treatment) to 5.6 ± 1.2% (*P* < 0.05; Tukey’s test) and decreased disease incidence from 21% to 5%. While Ba01 treatment at 5x10^6^ and 1x10^7^ CFU/mL did not reduce disease severity, disease incidence decreased from 21% to 7% and 8%, respectively (Fig 6B, 6C and 6D). Ba01 treatment did not stimulate plant growth or increase potato tuber yield (Fig 6E and 6F).

**Fig 7:**
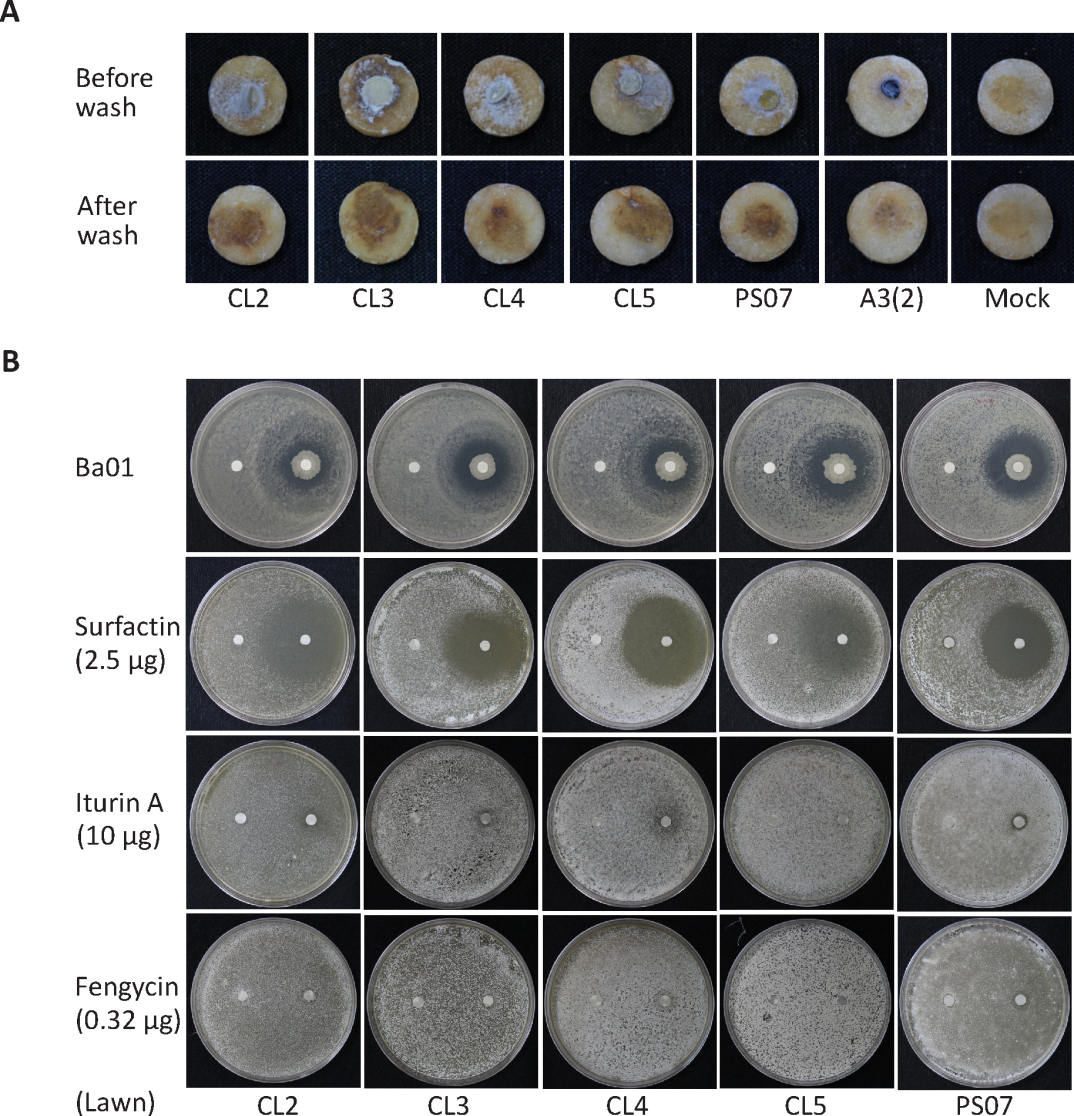
Ba01 inhibits the growth of multiple *S*. *scabies* strains isolated from the field trial. **A.** Potato slide assays were used to test the pathogenicity of *S*. *scabies* isolates. Agar disks with or without *S*. *scabies* spores were inverted onto potato tuber slices and incubated at 28°C for six days in the dark and photographed. Nonpathogenic *Streptomyces coelicolor* was used as a negative control, and a SSM1 agar disc was used as a mock control. **B.** Disc diffusion assays were used to test the anti-bacterial activity of Ba01 and three pure compounds against multiple *S*. *scabies* strains.

We isolated four *Streptomyces* isolates (CL2 to CL5) from scabby potato tubers in different experimental blocks and identified these strains as pathogenic *S*. *scabies* based on potato tuber slice assays (Fig 7A). Ba01 exhibited inhibitory effects toward four *S*. *scabies* isolates (CL2 to CL5), but these isolates were slightly less susceptible to Ba01 than PS07 based on disk diffusion assays. Interestingly, our data showed similar inhibitory activity of surfactin, iturin A, and fengycin against four *S*. *scabies* strains (CL2 to CL5) isolated from the field and *S*. *scabies* PS07 (Fig 7B), indicating that differential tolerance of *S*. *scabies* strains to Ba01 might be due to specific characteristics of *S*. *scabies* and not to the secretion of surfactin, iturin A, or fengycin.

## Discussion

Biological control agents have been extensively studied to combat plant pathogens in order to reduce environmental pollution, ecological disturbance due to pesticides used in fumigation, and pre-sowing tuber/seed treatments. In this study, we tested six *Bacillus* isolates and found that *B*. *amyloliquefaciens* Ba01 exhibited stronger inhibitory effects than *B*. *subtilis* or *Bacillus* sp. against the potato common scab pathogen. Additionally, *S*. *scabies* hyphae in the undifferentiated zone were still undifferentiated after 20 days, indicating that the hyphal growth of *S*. *scabies* was inhibited and was not simply due to growth delay. To our knowledge, only two studies have investigated the use of *Bacillus* isolates (*Bacillus* sp. sunhua and *B*. *amyloliquefaciens* BAC03) to control this plant pathogen [19, 20]. Our findings that surfactin, iturin A, and fengycin acted as antibiotics against *S*. *scabies* are partially supported by studies in which Han and colleagues showed that iturin A was responsible for combating *Streptomyces* species. In addition, these three compounds, controlled by the *srf*, *bmy*, and *fen* genes, respectively, have been shown to be secreted from *B*. *amyloliquefaciens* FZB42 [32]. Although surfactin and fengycin were not identified by Han *et al*. to be secreted by *Bacillus* sp. sunhua, these compounds have been detected in studies against other pathogens [33]. In this study, we provide evidence that Ba01 secretes surfactin, iturin A, and fengycin based on IMS analysis, while the other peaks seen on the spectra are derivatives of surfactin, iturin, or fengycin. Based on this powerful IMS technique, we also determined the secreted amount of these three compounds, which will aid in the investigation of which compound exerts the strongest effects. Ba01 produces a stronger inhibitory effect than any single pure compound (surfactin, iturin A, or fengycin), which indicates that compounds secreted from Ba01 might exert synergistic effects *S*. *scabies*. Further experiments may include disrupting a single gene (*srfA*D, *ituD*, or *fenA*), two genes (*srfA*D *ituD*, *srfA*D *fenA*, or *ituD fenA*), or three genes (*srfA*D *ituD fenA*) from the Ba01 isolate and testing the ability of these mutants to inhibit *S*. *scabies* growth and sporulation or their biocontrol efficacy against potato common scab in pot assays and field trials. Such experiments will provide additional evidence to show that synthesis of these compounds is required to inhibit the growth of *S*. *scabies* and reduce scab symptoms. However, several attempts to obtain these mutants via homologous recombination or in-frame deletion strategies were unsuccessful in the Ba01 isolate (S1 File), possibly due to the ‘wild’ nature of Ba01 (ie, low level of genetic competence and transformation amenability). Similar disruption techniques were successful in *B*. *amyloliquefaciens* FZB42 (data not shown), indicating that an efficient gene deletion is dependent on a specific isolate. In the future, disruption methods will need further modifications to disrupt genes in the Ba01 isolate.

We tested whether surfactin, iturin A, and fengycin exhibited synergistic effects by determining the FIC, but did not observe synergistic activity between any two compounds (Table 2). Because the endpoint of FIC requires complete inhibition, the combination of any two compounds might partially inhibit growth but does not reach complete inhibition. Meanwhile, these results may also indicate that other compounds or proteins secreted from Ba01 may enhance the effects of surfactin, iturin A, or fengycin on *S*. *scabies* growth suppression. Previous studies showed that *Bacillus* species secrete compounds such as macrolactin A, bacillaene, and difficidin in the presence of plant pathogens [33–35]. Therefore, additional studies to identify other secondary metabolites or proteins secreted from *B*. *amyloliquefaciens* are warranted. Interestingly, several studies have demonstrated that *B*. *amyloliquefaciens* not only can suppress diseases but also promote plant growth [20, 36]. Nevertheless, in this study Ba01 only suppressed scab symptoms and did not promote potato plant growth or increase tuber weight.

In addition to secreting antibacterial compounds against *S*. *scabies*, it is possible that Ba01 elicits induced systemic resistance (ISR) of the potato plant. Previous studies have shown that *Bacillus* isolates can elicit ISR of various plant hosts, including tomato, bell pepper, muskmelon, watermelon, sugar beet, tobacco, cucumber, and loblolly pine, to combat pathogens [37–39]. For example, Chowdhury and colleagues found that cyclic lipopeptides and volatiles produced by *B*. *amyloliquefaciens* FZB42 can trigger ISR pathways and protect plants against pathogens [40]. Future studies involving pot experiments and field trials can address whether Ba01 triggers ISR signaling.

The less potent inhibitory effects of Ba01 against potato common scab in the field trial than those in the pot assays may be due to the complicated microbial community in the soil of the field trial. We isolated several *S*. *scabies* isolates from scabby potato tubers in the field and found that these isolates were pathogenic in potato tuber slice assays. However, these *S*. *scabies* isolates were less susceptible to Ba01 than *S*. *scabies* PS07, which was used in the pot assays, suggesting that *S*. *scabies* isolates in the field were relatively tolerant to Ba01 and that Ba01 demonstrates differential inhibitory effects against various *S*. *scabies* isolates. In the future, we may consider combining two or more *B*. *amyloliquefaciens* isolates in order to control multiple *Streptomyces* isolates in the field. Meanwhile, additional field tests with diverse moisture, temperature, soil pH, and environmental conditions can be conducted in order to test the efficacy of Ba01 in various situations. Although the Ba01 isolate can reduce disease severity in both pot assays and field trials, the application of Ba01 on potato plants via pouring of Ba01 solution requires extensive labor. This application strategy is challenging to farmers who grow potatoes due to the limited availability of manpower. Alternatively, an agricultural machine with an automatic pouring system can be developed to replace human workers, or the Ba01 isolate could be mixed with soil.

In summary, we present a report that *B*. *amyloliquefaciens* reduces symptoms of naturally occurring potato common scab. The potential mechanisms by which Ba01 inhibits the growth of *S*. *scabies* at least in part are through the secretion of surfactin, iturin A, or fengycin. The evidence that Ba01 inhibits the growth and sporulation of *S*. *scabies* and reduces scab symptoms in pot assays and field trials suggests that Ba01 is a potential biocontrol agent for controlling potato common scab.

## Supporting information

S1 File Attempts to construct *B. amyloliquefaciens* mutants. (PDF)
