# Supplementary material for: Biological control of potato common scab by Bacillus amyloliquefaciens Ba01
Source: PLoS One. 2018 Apr 26;13(4):e0196520. doi: 10.1371/journal.pone.0196520 (PMC5919641; doi:10.1371/journal.pone.0196520)
Supplement: S1 File — (PDF) [file pone.0196520.s001.pdf]

Supporting information

Materials and Methods

Attempts to obtain mutants via homologous recombination

1. Construction of *srfA*D, *ituD,* and *fenA* disruption cassettes

We attempted to generate *Bacillus amyloliquefaciens* Ba01 deletion mutants by homologous recombination. To disrupt the surfactin synthesis gene *srfA*D, approximately 1 kb of the 5' and 3' noncoding regions (NCRs) of the *srfA*D ORF were PCR amplified with primers JC1548/JC1549 (for the 5' NCR) or JC1551/JC1552 (for the 3' NCR) from Ba01 genomic DNA. The 5' and 3' NCR PCR products of the *srfA*D gene were digested with the restriction enzymes BspEI and BamHI/HindIII, respectively. The BamHI/HindIII-digested PCR products of the 3' *srfA*D^{NCR} were purified using the Gel/PCR DNA Isolation System kit (Viogene, Taiwan) and then cloned into plasmid pMiniMAD (which carries the erythromycin resistance gene Erm^R; Table B), resulting in plasmid pCL13 (Table B). The BspEI-digested 5' *srfA*D^{NCR} PCR product was purified and cloned into pCL13 to create the *srfA*D disruption plasmid pCL21 (Table B). The pCL21 plasmid was PCR amplified with primers JC1548/JC1552 to obtain the 5' *srfA*D^{NCR}-Erm^R-3' *srfA*D^{NCR} disruption cassette to transform into Ba01.

A similar approach was used to disrupt the iturin A synthesis gene *ituD*. To disrupt

ituD, approximately 1 kb of the 5' and 3' NCRs of the *ituD* ORF were PCR amplified with primers JC1553/JC1554 (for the 5' NCR) or JC1555/JC1556 (for the 3' NCR) from Ba01 genomic DNA. The 5' and 3' NCR PCR products of the *ituD* gene were digested with the restriction enzymes BspEI and BamHI/HindIII, respectively. The BamHI/HindIII-digested PCR product of 3' $ituD^{NCR}$ was purified using the Gel/PCR DNA Isolation System kit and then cloned into the plasmid pMiniMAD, resulting in plasmid pCL15 (Table B). The BspEI-digested 5' $ituD^{NCR}$ PCR product was purified and cloned into pCL15 to create the *ituD* disruption plasmid pCL19 (Table B). The pCL19 plasmids were PCR amplified with primers JC1550/JC1556 to obtain the 5' $ituD^{NCR}$-Erm^{R}-3' $ituD^{NCR}$ disruption cassette to transform into Ba01.

To disrupt the fengycin synthesis gene *fenA*, approximately 1 kb of the 5' and 3' NCRs of the *fenA* ORF were PCR amplified with primers JC1557/JC1558 (for the 5' NCR) and JC1559/JC1560 (for the 3' NCR) from Ba01 genomic DNA. The 5' and 3' NCR PCR product of the *fenA* gene was digested with the restriction enzymes BspEI and EcoRI/HindIII, respectively. The EcoRI/HindIII-digested PCR product of 3' $fenA^{NCR}$ was purified using the Gel/PCR DNA Isolation System kit and then cloned into the plasmid pMiniMAD, resulting in plasmid pCL18 (Table B). The BspEI-digested 5' $fenA^{NCR}$ PCR product was purified and cloned into pCL18 to create the *fenA* disruption plasmid pCL23 (Table B). The pCL23 plasmid was PCR amplified with primers

JC1557/JC1560 to obtain the 5’ *fenA*^{NCR}-*Erm*^{R}-3’ *fenA*^{NCR} disruption cassette to transform into Ba01.

2. Transformation of *B. amyloliquefaciens* Ba01

For the competence assay, Ba01 was grown in 2 mL of 1X modified competence (MC) medium supplemented with 20 μL of 300 mM $MgSO_4$ for 4.5 h at 37°C. MC medium (1X) was made with a solution containing 100 mM K_2HPO_4, 100 mM KH_2PO_4, 2% glucose, 22 mg/L ferric ammonium citrate, 3 mM trisodium citrate, 0.1% casein hydrolysate, and 0.2% potassium glutamate. Then, 3 μg of the disruption cassette were added into 400 μL of the culture in an eppendorf tube. The culture was grown an additional 1.5 h at 37°C and plated onto LB containing 1 μg/mL erythromycin and 25 μg/mL lincomycin and grown for three days at 28°C. Colony PCR was used to analyze *srfA*D, *ituD,* and *fenA* mutants with primers JC1468/JC1469, JC1466/JC1467, or JC1470/JC1471, respectively [1].

Attempts to obtain mutants via an in-frame deletion strategy

1. Construction of *srfA*D, *ituD,* and *fenA* disruption cassettes

To disrupt the *srfA*D gene, approximately 1 kb of the 5’ and 3’ NCRs of the *srfA*D ORF was PCR amplified with primers JC1458/JC1459 (for the 5’ NCR) or

JC1460/JC1461 (for the 3’ NCR) from Ba01 genomic DNA. The 5’ and 3’ NCR PCR products of the *srfA*D gene were digested with two restriction enzymes (BamHI/XhoI and XhoI/KpnI, respectively). The two fragments were simultaneously ligated with the BamHI and KpnI sites of pMiniMAD to generate pCL7.

A similar approach was used to disrupt the *ituD* gene. To disrupt the *ituD* gene, approximately 1 kb of the 5’ and 3’ NCRs of the *ituD* ORF was PCR amplified with primers JC1454/JC1455 (for the 5’ NCR) or JC1456/JC1457 (for the 3’ NCR) from Ba01 genomic DNA. The 5’ and 3’ NCR PCR products of the *ituD* gene were each digested with two restriction enzymes (HindIII/XhoI and XhoI/KpnI, respectively). The two fragments were simultaneously ligated with the HindIII and KpnI sites of pMiniMAD to generate pCL9.

A similar approach was used to disrupt the *fenA* gene. To disrupt the *fenA* gene, approximately 1 kb of the 5’ and 3’ NCR of the *fenA* ORF was PCR amplified with primers JC1462/JC1463 (for the 5’ NCR) or JC1464/JC1465 (for the 3’ NCR) from Ba01 genomic DNA. The 5’ and 3’ NCR PCR products of the *fenA* gene were digested with two restriction enzymes (BamHI/XhoI and XhoI/HindIII, respectively). The two fragments were simultaneously ligated with the BamHI and HindIII sites of pMiniMAD to generate pCL11.

2. Transformation of *B. amyloliquefaciens* Ba01

We introduced 3 μg of plasmid pCL7 into Ba01 by transformation as described above, and two colonies were chosen to grow in 3 mL LB liquid medium containing 1 μg/mL erythromycin and 25 μg/mL lincomycin overnight at 28°C for plasmid replication. The cultures were serially diluted and plated onto LB medium containing erythromycin and lincomycin overnight at 37°C (restrictive temperature for plasmid replication). To evict the integrated plasmid, three colonies were incubated in 3 mL LB broth for 12 h at 25°C and subcultured 1:100 in fresh LB broth. The cultures were incubated at 25°C for an additional 12 h. Subcultures were repeated two more times, and the final serially diluted solution was plated onto LB medium at 37°C overnight. Colonies were patched onto LB plates and LB containing erythromycin and lincomycin, and drug-sensitive colonies representing potential mutants were chosen to conduct colony PCR with primers JC1468/JC1469, JC1466/JC1467, or JC1470/JC1471 for *srfA*D, *ituD,* or *fenA* ORFs, respectively.

Table A. PCR primers used in this study.

Primer	Use	Sequence (5’-> 3’)
fD1	16s rRNA	AGAGTTTGATCCTGGCTCAG
rP2	16s rRNA	ACGGCTACCTTGTTACGACTT
p-*gyr*A-F	*gyrA*	CAGTCAGGAAATGCGTACGTCCTT
p-*gyr*A-R	*gyrA*	CAAGGTAATGCTCCAGGCATTGCT
atpDPF	*atpD*	GTCGGCGACTTCACCAAGGGCAAGGTGTTCAACACC
atpDPR	*atpD*	GTGAACTGCTTGGCGACGTGGGTGTTCTGGGACAGGAA
JC1458	5’ NCR of *srfAD* (BamHI)	aggagggatccCGGCGAAAGAATGGATCGGG
JC1459	5’ NCR of *srfAD* (XhoI)	aggagctcgagCTGGACCATTGGCGGGCTTC
JC1460	3’ NCR of *srfAD* (XhoI)	aggagctcgagTTGTTATAGGATATGACAGACAGC
JC1461	3’ NCR of *srfAD* (KpnI)	aggagggtaccCGCGTAATTTTCCTTCGTC
JC1454	5’ NCR of *ituD* (HindIII)	aggagaagcttCGTAAACATTCAAAATGGCGGA
JC1455	5’ NCR of *ituD* (XhoI)	aggagctcgagTTAAAATAAAGCGCCCAGGA
JC1456	3’ NCR of *ituD* (XhoI)	aggagctcgagATTGTTCATGAGATTCCTCC
JC1457	3’ NCR of *ituD* (KpnI)	aggagggtaccTCAACGGACTGATCGGTTTT
JC1462	5’ NCR of *fenA* (BamHI)	aggagggatccCCGTCTGAACGTCCTAGCCA
JC1463	5’ NCR of *fenA* (XhoI)	aggagctcgagGAAAGCATGGTCGGCGTGCT
JC1464	3’ NCR of *fenA* (XhoI)	aggagctcgagGTTCTTCAATGGAATCCCTCC
JC1465	3’ NCR of *fenA* (HindIII)	aggagaagctt CGGACATCCATGCCTCTTTC
JC1468	*srfAD* ORF	aaaCCGCCGTTGAGGATTTTGAA
JC1469	*srfAD* ORF	aaaCATGTGGCCGTCCGAAAACT
JC1466	*ituD* ORF	aaaAGTGTATGCCGCACCCTTTT
JC1467	*ituD* ORF	aaaGAGCGATGCGATCTCCTTGG
JC1470	*fenA* ORF	aaaGCGAGAGGCTGGTATTGCAT
JC1471	*fenA* ORF	aaaGAACACCTTTCACTGGCGGA
JC1548	5’ NCR of *srfAD* (BspEI)	aaatccggaGTTAACGACAAACGGGAAGG
JC1549	5’ NCR of *srfAD* (BspEI)	aaatccggaCTGGACCATTGGCGGGCTTC
JC1550	Check direction of 5’ NCR	AGACAATCTCCCGTCCTCTGTT
JC1551	3’ NCR of *srfAD* (BamHI)	aaaggatccTTGTTATAGGATATGACAGACAGC
JC1552	3’ NCR of s*rfAD* (HindIII)	aaaaagcttCGCGTAATTTTCCTTCGTC
JC1553	5’ NCR of *ituD* (BspEI)	aaatccggaCGTAAACATTCAAAATGGCGGA
JC1554	5’ NCR of *ituD* (BspEI)	aaatccggaTTAAAATAAAGCGCCCAGGA

JC1555	3' NCR of *ituD* (BamHI)	aaaggatccATTGTTCATGAGATTCCTCC
JC1556	3' NCR of *ituD* (HindIII)	aaaaagcttTCAACGGACTGATCGGTTTT
JC1557	5' NCR of *fenA* (BspEI)	aaatccggaCCGGGCGAAGATGTCTTGTA
JC1558	5' NCR of *fenA* (BspEI)	aaatccggaTGCAAGGGCAGTTTCCGTTA
JC1559	3' NCR of *fenA* (EcoRI)	aaagaattcGTTCTTCAATGGAATCCCTCC
JC1560	3' NCR of *fenA* (HindIII)	aaaaagcttAGAAAAGTGGTACCCGGCTT

*Lowercase letters represent restriction enzyme cutting sites, including adenine nucleotides to protect primers.

Table B. Plasmids used in this study.

Plasmid	Relevant insert	Parent	Strategy
pMiniMad		[2]	
pCL13	3'NCR of *srfA*D	pMiniMad	Homologous recombination
pCL21	5'NCR of *srfA*D	pCL13	Homologous recombination
pCL15	3'NCR of *ituD*	pMiniMad	Homologous recombination
pCL19	5'NCR of *ituD*	pCL15	Homologous recombination
pCL18	3'NCR of *fenA*	pMiniMad	Homologous recombination
pCL23	5'NCR of *fenA*	pCL18	Homologous recombination
pCL9	3'NCR and 5'NCR of *srfA*D	pMiniMad	In-frame deletion
pCL7	3'NCR and 5'NCR of *ituD*	pMiniMad	In-frame deletion
pCL11	3'NCR and 5'NCR of *fenA*	pMiniMad	In-frame deletion

References

1. Konkol MA, Blair KM, Kearns DB. Plasmid-encoded ComI inhibits competence in the ancestral 3610 strain of *Bacillus subtilis*. J Bacteriol. 2013;195(18):4085-93.
2. Patrick JE, Kearns DB. MinJ (YvjD) is a topological determinant of cell division in *Bacillus subtilis*. Mol Microbiol. 2008;70(5):1166-79.
